# Novel risk patterns of vasovagal reactions in NZ blood donations complicated by COVID-19 restrictions

**DOI:** 10.3389/fpubh.2023.1180279

**Published:** 2023-05-25

**Authors:** Wen-Hua Wei, Meredith Smith, Amber Vera, Kelly Meads, Jillayne Hessell, Laura Reid, Lisa Scott, Asuka Burge, Susy Kirwan, Richard Charlewood, Deepak Sadani, Deborah Walkden, Anup Chand

**Affiliations:** ^1^New Zealand Blood Service, Auckland, New Zealand; ^2^Centre for Biostatistics, Division of Population Health, Health Services Research and Primary Care, The University of Manchester, Manchester, United Kingdom

**Keywords:** blood donation, COVID-19, donor adverse reaction, immediate vasovagal reaction, interaction, vasovagal reaction

## Abstract

**Introduction:**

Vasovagal reactions (VVRs) are common but complex donor adverse reactions (DAEs) in blood donations. VVRs have been extensively studied with a multitude of risk factors identified including young age, female gender and first-time donor status. How they may interplay remains obscure.

**Methods:**

A total of 1,984,116 blood donations and 27,952 immediate VVRs (iVVRs) and 1,365 delayed VVRs (dVVRs) reported between 2011 and 2021 in NZ were used in multivariate logistic regression analyses each concerning donations with iVVRs as cases and those free of DAEs as controls. For each analysis stepwise selection was used to identify the best model and risk factors carrying significant main effects and/or interactions. Identified interactions informed further in-depth regression analyses to dissect iVVR risk patterns.

**Results:**

Over 95% of VVRs were iVVRs that had lower female preponderance and deferrals than dVVRs. iVVRs had a school seasonal pattern in whole blood donations driven by first-time donors from schools/colleges, and interactions between gender and age group differentiating the first-time from repeat donations. Subsequent regression analyses identified the known and novel risk factors of year and mobile collection sites and their interactions. iVVR rates were roundly elevated in 2020 and 2021 probably because of COVID-19 restrictions like facemask wearing. Exclusion of the 2020 and 2021 data removed the interactions with year, but confirmed interactions of gender with mobile collection sites (*p* = 6.2e-07) in first-time donations only and with age group in repeat donations only (*p* < 2.2e-16), together indicating young female donors at the highest risk of iVVRs. Our results also revealed that donation policy changes contributed to the year effects; donors had a lower iVVR risk at mobile sites than well-medicalized donation centers probably because of under-reporting.

**Conclusion:**

Modeling statistical interactions is valuable in identifying odds and revealing novel iVVR risk patterns and insights into blood donations.

## Introduction

Vasovagal reactions (VVRs) are the most common but complex donor adverse events (DAEs) in blood donations (1, 2). During a VVR, donors may experience a drop in arterial blood pressure and cerebral perfusion leading to pre-syncopal symptoms (e.g., dizziness) or even vasovagal syncope (i.e., loss of consciousness) (3). Vasovagal syncope, while being considered to be clinically severe, accounts for only a small proportion (e.g., < 1%) of VVRs, although reliable estimates are lacking because severity assessment is often optional (1, 4, 5). VVRs can be triggered by psychological (e.g., fear of seeing blood) (5) and/or physiological (e.g., losing a substantial volume of blood) stimuli. They cause donor discomfort and safety concerns, therefore negatively impacting donor return rates, donation operational management (2, 4) and overall performance.

VVR incidence rates per 1,000 donations vary widely, e.g., from 10 to 125 in whole blood (WB) donations and 1.6 to 41.7 in apheresis collections (6, 7). Such wide variations reflect substantial heterogeneity in donation operations and VVR reporting (e.g., not all VVRs got reported) across haemovigilance systems. To address the challenges in benchmarking and comparison, the International Haemovigilance Network developed a database aggregating data from 24 haemovigilance systems but reported median VVR rates as low as 3.4 and 1.5 for WB and apheresis donations, respectively, (1), highlighting that much more work is needed.

A VVR can be further classified as immediate (iVVR) if it happens at a collection site or delayed (dVVR) if it happens beyond the collection site and within 24 h following donation. Although dVVRs account for only a small proportion (~10%) of the total VVRs reported, this could be an under-estimate as donors might not necessarily report symptoms after leaving the collection site (8, 9).

VVRs have been extensively studied globally, with a multitude of risk factors identified (2, 8, 10). Commonly observable factors include young age, female gender, and first-time donation status (7, 11–13), indicating they may carry independent risk of VVRs (14). For example, donors as young as 16-to 17-years-old were significantly more likely to experience VVRs in whole blood donations than older donors (14). Other risk factors include anthropometric measures such as low body mass index and low estimated blood volume (15), psychological factors like fear of blood drawn and pain (15, 16), contextual factors such as inexperienced phlebotomists (17, 18) and collection site settings related to long waiting and/or bleeding time (19). Intriguingly, previously assumed genetic risk factors such as history (20) particularly familial history (21) of vasovagal syncope have received renewed interest (10, 22), and may add a missing piece of the puzzle of VVR causal mechanisms that however remain largely obscure. Therefore, implementation of VVR intervention and prevention measures [e.g., pre-donation water loading and applied muscle tension (2)] would be sensible and effective approaches at this stage (23, 24).

Further research is needed to address a few shortfalls. Firstly, major donation environmental changes like implementation of interventions (24) and the COVID-19 pandemic (25) could influence risk patterns of VVRs, which is yet to be assessed. Secondly, the identified VVR risk factors may not only carry additive main effects but also interact with each other (15, 16) yielding non-additive interactive effects that are yet to be characterized. Such statistical interactions would illuminate how VVR risk factors might interplay. Thirdly, large-scale integrative analyses are needed (2) to systematically assess risk factors and their interactions in one model and thus generate overviews of VVR risk patterns under different circumstances, to inform donation practices.

Here we used a comprehensive dataset of blood donations and DAEs collated by the New Zealand Blood Service (NZBS) to study VVR risk patterns in NZ blood donors. We first characterized blood donations and VVRs by known risk factors, as well as by year and type of collection sites. Concentrating on iVVRs, we then performed multivariate logistic regression analyses, modeling main effects and interactions together for first-time and repeat donations separately. Identified interactions were examined closely and used to inform in-depth analyses to dissect risk patterns of iVVRs and identify the most vulnerable donors.

## Materials and methods

### Study participants and measures

This is a retrospective study of blood donations and DAEs between 2011 and 2021 in NZ. NZBS is the sole provider of blood and blood products in NZ and is responsible for the collection of blood from volunteer non-remunerated donors and to the provision of blood products within the hospital environment. NZBS has collected DAE data reported by nurses in charge using a standard form based on guidance and definitions for complications related to blood donation by the International Society of Blood Transfusion.^1^ DAE reports received were reviewed by medical officers and revised if necessary to ensure accurate classifications and then entered into dedicated secure databases.

Data from blood donations between 2011 and 2021, including donor gender, donation type (either WB, or plasmapheresis, or plateletpheresis), donation date, age at donation, donation site and site type (i.e., mobility as either fixed or mobile), first-time donor status, were extracted from dedicated NZBS databases. DAEs reported between 2011 and 2021 were extracted from a NZBS Haemovigilance database, including the same fields for blood donations above plus fields storing DAE categories, symptoms and donor outcome. Only DAEs related to either WB donations, or plasmapheresis or plateletpheresis were concerned in this study. Data quality control of DAEs removed records with missing entries of symptoms (*n* = 28), or with a wrong flag of “Citrate Reaction” in WB donations (*n* = 26), or with a duplicate entry of red cells not returned (*n* = 13,913), giving a rate of exclusion of initial data of 18.9%. Further, entries of the first-time donation flag of each DAE were checked against that recorded in the database of blood donations and corrected accordingly if a mismatch was detected. In total, nearly 2 million (1,984,116) blood donations and 60,028 DAEs (including 27,952 iVVRs and 1,365 dVVRs) during the study period were analyzed.

This is a minimal risk observational study utilizing only de-identified donor data and thus requires neither ethical approval nor written informed consent from patients or patient guardians, according to the Standard Operating Procedures for the Health and Disability Ethics Committees of NZ. The study is approved by the Clinical Advisory Group (i.e., institutional review board) of NZBS. All research has conformed to the principles embodied in the Declaration of Helsinki.

### Data analysis

Blood donation and DAE data were first characterized by known risk factors of VVR such as gender, age, first-time donation status, type of blood donations, and type of collection sites. Seasonal and yearly impact were also explored. Chi-square tests were used for group comparisons between iVVR and dVVR. DAE or iVVR rates were calculated as per 1,000 donations as appropriate. Following previous studies (26) and donor recruitment experience (e.g., student first-time donors likely to be younger than 22), three donation age groups were defined: age < =22 years, 22 < age < =40 and age > 40, and used in subsequent analyses focusing on iVVRs.

The blood donation data were merged with the DAE data into a combined list with additional fields storing whether each donation was associated with a DAE and what type of DAEs if yes. In the combined list, donations associated with DAEs other than iVVRs were first excluded, and then donations associated with iVVRs were set as cases and those free of DAEs were set as controls. The resultant list was further split into a list of only first-time donations with/without iVVR and a list of the remaining donations by repeat donors. Statistical analyses were performed using R (27) (v4.1.1) and R packages *MASS*, *stats*, *DescTools*, *ggplot2*, and *questionr*. Multivariate logistic regression analyses were performed for first-time donations and repeat donations separately. The regression models concerned gender, age group, type of donation site, year, and their interactions for convenience of comparisons, with donation site and donation type excluded because of data sparsity at certain levels of either of the two variables. For each set of logistic regression analyses, a three-step approach was taken using the *glm()* function with the regression family parameter set as binomial and the *stepAIC()* function: (1) fitting the NULL model where all variables of interest without any interactions were fitted in a logistic regression model; (2) fitting the FULL model where all variables of interest and their all possible interactions were fitted in a logistic regression model; (3) the results of the NULL and the FULL models were fitted into the *stepAIC()* function with the direction parameter set as ‘both’ to perform stepwise selection of the best model explaining the most phenotypic variance. The resultant best model concerned only covariates (i.e., variables and/or their interactions) with significant effects in the regression, and was further analyzed using the *anova()* function to quantify variance explained by each covariate and examine significance using the built-in Chi-square test. The *odds. Ratio()* function of the *questionr* package was used to calculate odds ratios and test statistical significance using a built-in Fisher’s exact test.

## Results

NZ blood donations and DAEs reported between 2011 and 2021 are summarized in Table 1. Among the substantial changes sketched by Sparklines, the most striking ones were the sharp increases of DAEs in 2020 and 2021 coincident with the COVID-19 pandemic and the surging demand of plasmapheresis in recent 5 years or so. However, VVR remain the most common type of DAEs, with a proportion fluctuating around 50% of the total DAEs over the years (Table 1).

**Table 1:** Annual profiles of NZ blood donations and donor adverse reactions between 2011 and 2021*.

	2011	2012	2013	2014	2015	2016	2017	2018	2019	2020	2021	Sparkline
Donor	92,826	89,214	80,976	79,257	79,651	79,070	76,406	78,084	80,529	80,800	85,501	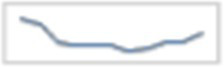
WB donation	147,093	139,845	125,684	120,668	119,554	114,779	111,189	112,162	113,329	113,699	115,315	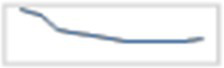
Plasma donation	28,886	30,179	29,585	38,099	46,983	54,059	54,125	59,895	65,192	93,669	108,669	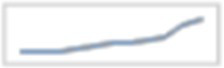
Platelet donation	6,491	6,527	4,942	3,570	3,377	2,878	2,766	2,648	2,682	2,823	2,754	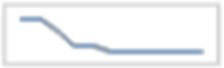
DAE	4,294	4,767	4,470	4,414	4,453	4,651	5,250	5,960	5,623	7,471	8,675	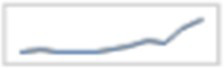
VVR%	54.5%	47.7%	47.7%	50.5%	51.1%	52.4%	47.0%	46.6%	47.8%	46.0%	49.0%	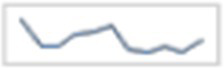
VVR rate	12.8	12.9	13.3	13.7	13.4	14.2	14.7	15.9	14.8	16.4	18.8	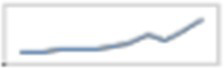

* WB, whole blood; DAE, donor adverse reaction; VVR%, percent of vasovagal reactions out of DAEs; VVR rate, VVRs per 1000 donations.

Over 95% of VVRs were immediate that differed from the delayed significantly in deferral (*p* = 0.01), permanent deferral (*p* = 4.2e-07), and female preponderance (*p* = 7.1e-10) (Supplementary Table S1). About 21% (292 out of 1,365) of dVVRs happened in first-time donors, which was less frequently than that of iVVRs (~33%). First-time donors with dVVRs were deferred at a rate of nearly 10% (3.4% + 6.5%), of which most were permanent deferrals dominated by female donors (i.e., 18 out of a total of 19). On the other hand, repeat donors who were permanently deferred because of dVVRs (i.e., 84–19 = 65) tended to be much older than those permanently deferred because of iVVRs (Supplementary Table S1). Further investigation is required to better understand dVVRs and the differences from iVVRs.

**Figure 1:**
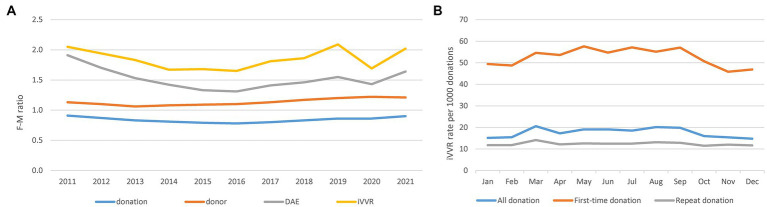
Gender differences in blood donations, donors and DAEs and school seasonal patterns of iVVRs in whole blood donations. **(A)** Gender differences in F-M ratio calculated as the number of females divided by the number of males per year per category (left). **(B)** School seasonal patterns of monthly iVVR rates in whole blood donations (right).

Concentrating on iVVRs hereafter, we first observed clear gender and school seasonal patterns (Figure 1). Comparing to male donors, female donors slightly outnumbered and donated slightly less frequently but were clearly more likely to experience DAEs, particularly VVRs over the years (Figure 1A). Considering WB donations only, the monthly iVVR rates showed a clear school seasonal pattern in all and the first-time donations but not in the repeat donations (Figure 1B), indicating that the pattern was probably driven by student donors available mainly at school/college terms between March and October in NZ. The iVVR rate was also the highest in March in the repeat donations but the difference from the remaining months was small (Figure 1B).

**Figure 2:**
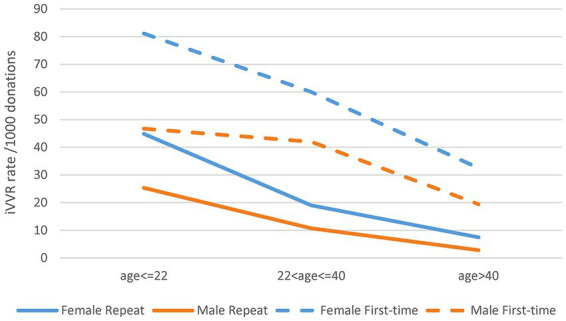
Statistical interactions between gender and age group in the first-time and repeat donations. The top two gender lines of the first-time donations were not in parallel between the first two age groups indicating certain interactions; the bottom two gender lines of the repeat donations were not in parallel all the way toward a crossing point indicating strong interactions.

We then observed statistical interactions between age group and gender in iVVR rates in both the first-time and repeat donations (Figure 2). Clearly, iVVR rates in female donors, or the age < =22 group or the first-time donations, were always higher than their counterparts, respectively. However, iVVR rates in female donors reduced more sharply than that in male donors from the age < =22 to the 22 < age < =40 group, and then at a similar level as males toward the age > 40 group, indicating patterns of interactions (i.e., lines were not in parallel). These results also indicated that the first-time and repeat donations should be analyzed separately.

Considering donations with iVVRs as cases and those free from DAEs as controls, multivariate logistic regression analyses detected significant interactions for both the first-time and repeat donations (Supplementary Table S2). Considering the first-time donations, significant risk factors were age group, gender, year, mobility, as well as interactions between mobility and year, age group and year, gender and mobility, gender and age group. These significant factors jointly explained only 2.8% of the total iVVR variance, of which ~0.2% were by interactions alone. For the repeat donations, significant risk factors included most of those aforementioned (except for mobility and its interaction with gender) and additional interactions between gender and year, age group and mobility, and among three factors of gender, age group and year, which jointly explained ~6.4% of the total iVVR variance (~0.2% by interactions alone) (Supplementary Table S2). Exclusion of the 2020 and 2021 data from the regression analyses removed most interactions with year (Supplementary Table S3), confirming that the 2 years of COVID-19 pandemic were indeed the triggers of statistical interactions. In contrast to Figure 2, a further exclusion of donations by donors younger than 18 years clearly changed the patterns of interactions between gender and age group in both the first-time and the repeat donations (Supplementary Figure S1).

**Figure 3:**
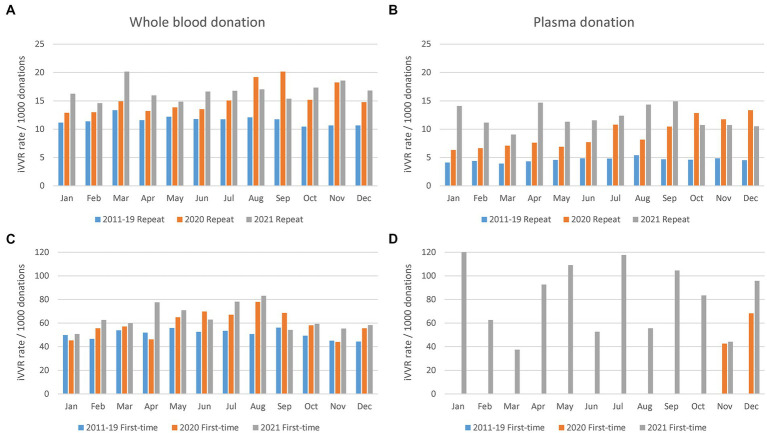
Monthly iVVR rates in 2020 and 2021 in contrast to the historic counterparts calculated using data between 2011 and 2019. **(A)** Repeat whole blood donations (top left). **(B)** Repeat plasma donations (top right). **(C)** First-time whole blood donations (bottom left). **(D)** First-time plasma donations (bottom right); 2011–19 Repeat (2020 Repeat, 2021 Repeat): Repeat donations between 2011 and 2019 (2020, 2021); 2011–19 First-time (2020 First-time, 2021 First-time): First-time donations between 2011 and 2019 (2020, 2021); First-time plasmapheresis donations started from November 2020.

Monthly iVVR rates in 2020 and 2021 were therefore compared against the historic counterparts calculated using data between 2011 and 2019 (Figure 3). In WB donations, historic iVVR rates in the repeat (Figure 3A) and first-time (Figure 3C) donations resembled the corresponding patterns presented in Figure 1B. In plasma donations however, monthly historic iVVR rates were consistent in repeat donations (Figure 3B) and were about 50% lower than the counterparts in WB donations, but were absent in first-time donations (Figure 3D) because NZBS started plasmapheresis of first-time donors from November 2020. In contrast to the historic counterparts, monthly iVVR rates clearly elevated in most 2020 and 2021 regardless of donation type, highlighting systematic changes in donation settings probably triggered by the COVID-19 pandemic such as mask wearing and lockdowns. The systematic impact appeared to be stronger in plasmapheresis than WB donations possibly because of the continuously increased demand of plasmapheresis that requires longer duration than WB donation. Furthermore, the monthly changes in 2020 or 2021 lost the school seasonal patterns probably due to varied COVID-19 restrictions during the course. Nonetheless, the extreme high iVVR rates in the first-time plasma donors (Figure 3D) could be due to a relatively small number of such donations as denominators (i.e., 98 and 742 in 2020 and 2021 respectively).

**Figure 4:**
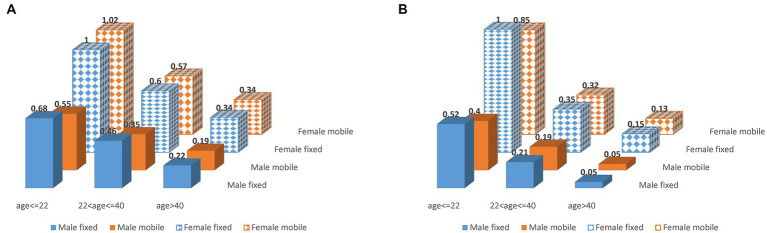
iVVR odds ratios by gender, age group and type of collection sites using data between 2011 and 2019. **(A)** First-time donations (left). **(B)** Repeat donations (right).

We further computed odd ratios of iVVRs using data between 2011 and 2019 in the first-time and repeat donations, respectively, (Figure 4; Supplementary Table S4). Clearly, young female donors had the highest iVVR risk in both cases. Intriguingly, female first-time donors shared similar levels of iVVR risk across the two types of collection sites, whereas male first-time donors had a lower iVVR risk at mobile sites than fixed sites with differences reduced by age groups (Figure 4A), which gave rise to the interactions between gender and mobility detected only in the first-time donations (*p* = 6.2e-07, Supplementary Table S3). In repeat donations however, iVVR odds ratios at mobile sites were consistently lower than counterparts at the fixed sites in both genders (equal in male donors at the age > 40 group), with differences reduced by age groups as well (Figure 4B).

## Discussion

Empowered by comprehensive data of NZ-wide blood donations, we closely examined key risk factors for iVVR and characterized their interactions for the first time. In addition to the well-known risk factors of first-time donation status, young age and female gender (2, 4, 13, 28), we identified calendar year and type of collection sites as additional risk factors, and a school seasonal pattern of iVVR rates in WB donations largely driven by student first-time donors available mainly in school terms. Furthermore, we detected statistical interactions between the identified risk factors (Figure 2; Supplementary Table S2) and used them to inform in-depth analyses leading to unique risk patterns differentiating the first-time from the repeat donations, and that differentiating donations prior to the COVID-19 pandemic from those during the pandemic where iVVR rates were roundly elevated in 2020 and 2021 probably because of various COVID-19 restrictions (Figure 3). Using data between 2011 and 2019 without any COVID-19 complication, we confirmed that young female donors were at the highest risk of iVVRs (14, 26) and observed that donors tended to have a lower (if not an equal) risk of iVVRs at mobile collection sites than fixed ones (Figure 4) for the first time.

The association of mobile sites with reduced iVVR risk appeared as a surprise initially as by a conventional perception it would the other way around. Since impact of type of collection sites on risk of iVVRs has been underexplored (7), it is not immediately clear what might drive the association. One possible reason could be under-reporting at mobile sites where donors seem less likely to report iVVR symptoms than in a well medicalized environment like donation centers. The only exception is that first-time female donors shared a similar level of iVVR risk across two types of sites probably because they are psychologically most vulnerable to iVVRs (7, 11–13) and topped iVVR rates across age groups as showed in Figures 2 and Supplementary Figure S1. Another possible reason could be that some mobile settings (e.g., open air) might somehow reduce pain sensitivity or increase tolerance of blood draw. There is evidence showing that pain sensitivity could change with environments (29). Further multidisciplinary research is warranted to investigate any underlying psychological and/or physiological mechanisms.

In addition to picking up odds, modeling statistical interactions provided new insights into NZ blood donations. After removal of the data during the Covid pandemic, highly significant interactions were still detected between gender and mobility in first-time donations (*p* = 6.2e-07) and between gender and age group in repeat donations (*p* < 2.2e-16) (Supplementary Table S3). The former interaction was present only in the first-time donations because of the special attributes of the first-time female donors as discussed above (Figure 4A), suggesting that mobile collection sites performed similarly as donation centers in NZ. The latter interaction defined a pattern of changes of iVVR risk with age differing female from male donors in repeat donations only (Figure 4), where young female donors were the trigger of the interaction. The latter interaction might become visually obvious after further removal of donations by donors younger than 18-year-old as illustrated in Supplementary Figure S1, where two gender lines were almost in parallel in first-time donations (i.e., no interaction) but not (moving toward a crossing point) in repeat donations (i.e., strong interaction). These results together reinforce the notion that young female donors ought to receive extra care regardless of the first-time status (14, 26). Marginally significant interactions of age group with type of collection sites and with year were also detected in repeat donations (Supplementary Table S3), which might indicate some operational issues discussed below.

Our results raised a couple of concerns about donor vigilance in NZ blood donations. First, only a small proportion of the total iVVR variance were explained by the risk factors identified in either the first-time donation model (i.e., <3%) or the repeat donation model (<7%), suggesting that some important variables might have been missed out from the current donor vigilance. Monitoring additional factors such as donor blood volume, fear and pain (15, 16) and improving phlebotomist training (18) would be very helpful. Second, the iVVR rates in NZ blood donations appeared to be similar to that in Australia (24) but considerably higher than some overseas counterparts (4, 13). Other than the unmonitored variables aforementioned, one obvious reason is the minimum age of donation in NZ was 16 years old – 2 years younger than that in the overseas peers (4, 13). Since these very young donors were subject to high risk of iVVRs as discussed above (14, 26), exclusion of donations by donors under 18 years old would dramatically reduce iVVR rates in the age < =22 group as illustrated in Supplementary Figure S1 in contrast to Figure 2. Other possible reasons include operational issues such as increased intake of new nurses/technicians, lack of consistency in following donation precautions like pre-hydration checking and using applied muscle tension.

In addition to the operational issues above, major changes to donation environment and operation policies could contribute to the significant year effects identified in both the first-time and the repeat donation models. One good example was the implementation of COVID-19 restriction measures in blood donation settings, of which facemask wearing could influence breathing, effective communication, and ability to detect early signs of adverse reactions (25, 30), generate hypercapnia or related issues, and subsequently elevate iVVR rates roundly (Figure 3). As a matter of fact, the COVID-19 pandemic itself could have generated certain anxiety to donors contributing to the increase of iVVR rates. Another good example is that after exclusion of donation data during the pandemic, the resultant regression coefficients from either the first-time or repeat donation model suggested consistently a significant increase of iVVRs from 2016 onwards. The timing coincided with a couple of NZBS donation policy changes in 2015: (1) stand down period following WB donation to apheresis donation was reduced from 60 days to 30 days from October; (2) stand down period following failure to return red cells during apheresis donation was reduced from 60 days to 30 days also from October. Nevertheless, further investigation is required to confirm if these policy changes might have triggered any negative impact on the iVVR risk.

Our study also highlighted a couple of research issues. First, fear and/or pain related psychological factors (15, 16), although remaining hidden from this study, likely influence first-time donors and young repeat donors pervasively as our results suggested (Figure 4). Such factors likely interact with the identified risk factors (31, 32) but probably in rather complicated ways that require well-designed large studies to tease out how they might interplay in triggering iVVRs. Second, while mechanisms regulating iVVR risk remain obscure, developing strategies for early screening and/or intervention (2, 24) can not only effectively mitigate iVVR risk but also help dissect the causal mechanisms by studying any genetic (10, 22) and/or epidemiological differences in iVVR cases that do not respond to interventions.

This study was limited by data available for comprehensive investigation. For example, we characterized that dVVRs were featured by female preponderance and high probability of permanent deferral (Supplementary Table S1), but were unable to investigate what caused the features due to a relatively small number of dVVRs reported during the study period. Further meta-analysis is needed to discover any roles of under-reporting (particularly from male donors) (8, 9) and better understand any special biological mechanisms underlying dVVRs. Besides, the issue of VVR under-reporting could limit accuracy of the identified risk patterns. Our analyses might benefit from classifying donations in additional smaller age groups for detailed pattern changes that are better addressed in future meta-analysis.

## Data availability statement

The data analyzed in this study is subject to the following licenses/restrictions: Applications for access to the de-identified data will be reviewed by the Clinical Advisory Group of NZ Blood Service. Requests to access these datasets should be directed to MS, haemo.vigilance@nzblood.co.nz.

## Ethics statement

Ethical review and approval was not required for the study on human participants in accordance with the local legislation and institutional requirements. Written informed consent from the participants’ legal guardian/next of kin was not required to participate in this study in accordance with the national legislation and the institutional requirements.

## Author contributions

W-HW, MS, RC, and AC conceived the idea of the study. W-HW, AC, MS, DS, and DW contributed to the design of the study and the acquisition, analysis or interpretation of the data. AV, KM, JH, LR, LS, AB, and SK contributed to interpretation of the results and discussion of clinical insights. W-HW performed the statistical analysis and prepared the first draft of the manuscript. AC, DW, DS, MS, RC, and SK revised it critically for important clinical content. W-HW and MS had full access to all the data in the study. All authors contributed to the content of the paper, reviewed and approved the final version and had responsibility for the decision to submit for publication.

## Conflict of interest

The authors declare that the research was conducted in the absence of any commercial or financial relationships that could be construed as a potential conflict of interest.

## Publisher’s note

All claims expressed in this article are solely those of the authors and do not necessarily represent those of their affiliated organizations, or those of the publisher, the editors and the reviewers. Any product that may be evaluated in this article, or claim that may be made by its manufacturer, is not guaranteed or endorsed by the publisher.

## Footnotes

^1^ https://www.isbtweb.org/resource/en2014isbtstandard-for-surveillance-of-of-complications-related-to-blood-donations.html
