# Supplementary material for: Novel risk patterns of vasovagal reactions in NZ blood donations complicated by COVID-19 restrictions
Source: Front Public Health. 2023 May 25;11:1180279. doi: 10.3389/fpubh.2023.1180279 (PMC10248232; doi:10.3389/fpubh.2023.1180279)
Supplement: Supplementary file 1 [file Data_Sheet_1.docx]

**Supplementary materials**

Figure S1: Statistical interactions between gender and age group in the first-time and repeat donations after exclusion of donations in 2020 and 2021 and by donors younger than 18 years old. The top two gender lines were almost in parallel indicating little interactions in first-time donations; the bottom two gender lines went towards a crossing point indicating significant interactions in repeat donations.

Table S1: Differences between immediate and delayed vasovagal reactions during the study period*

|  | Case | Deferral |  |  | Permanent |  |  |
| --- | --- | --- | --- | --- | --- | --- | --- |
|  |  | n (%Case) | F-M ratio | Age | n (%Case) | F-M ratio | age |
| iVVR^all^ | 27,952 | 1525 (5.5%) | 1.3 | 31.5±13.6 | 959 (3.4%) | 2.1 | 32.9±15.5 |
| dVVR^all^ | 1365 | 98 (7.2%) | 2.5 | 33.9±16.2 | 84 (6.2%) | 6.6 | 46.8±19.5 |
| iVVR^1st^ | 9238 | 257 (2.8%) | 2.0 | 23.3±9.5 | 233 (2.5%) | 1.7 | 28.4±12.9 |
| dVVR^1st^ | 292 | 10 (3.4%) | 4.0 | 20.5±5.7 | 19 (6.5%) | 18.0 | 29.1±17.3 |

*: Permanent: permanent deferral; iVVR^all^ (dVVR^all^) – all immediate (delayed) vasovagal reactions; iVVR^1st^ (dVVR^1st^) – immediate (delayed) vasovagal reactions in first-time donations

Table S2: Variance explained by significant risk factors of iVVR and/or their significant interactions using all data*

| Term | df | First-time |  | Repeat |  |
| --- | --- | --- | --- | --- | --- |
|  |  | %variance | p-value | %variance | p-value |
| Age_group | 2 | 1.502% | < 2.2e-16 | 4.312% | < 2.2e-16 |
| Gender | 1 | 0.752% | < 2.2e-16 | 1.462% | < 2.2e-16 |
| Year | 10 | 0.302% | < 2.2e-16 | 0.405% | < 2.2e-16 |
| Mobility | 1 | 0.069% | 1.6e-12 | 0.001% | 8.1e-02 |
| Mobility : Year | 10 | 0.070% | 2.0e-07 | 0.073% | < 2.2e-16 |
| Age_group : Year | 20 | 0.056% | 3.9e-03 | 0.032% | 2.7e-06 |
| Gender : Mobility | 1 | 0.045% | 9.3e-09 | n/a | n/a |
| Gender : Age_group | 2 | 0.015% | 3.6e-03 | 0.058% | < 2.2e-16 |
| Gender : Year | 10 | n/a | n/a | 0.017% | 3.4e-04 |
| Age_group : Mobility | 2 | n/a | n/a | 0.007% | 6.1e-04 |
| Gender : Age_group : Year | 20 | n/a | n/a | 0.021% | 4.7e-03 |

*: 9388 iVVR cases and 161,257 controls in logistic regression analysis of First-time donations; 17,334 cases and 1,767,930 controls in logistic regression analysis of Repeat donations; df: degree of freedom; %variance: percent of the total phenotypic variance explained; underlined p-value of mobility in the Repeat donation model indicating insignificant additive effects; interacting was unlined to show terms were separated by “:”; Age_group: 1 (age<=22), 2 (22<age<=40), 3 (age>40).

Table S3: Variance explained by significant risk factors of iVVR and/or their significant interactions using data between 2011 and 2019 *

| Term | df | First-time |  | Repeat |  |
| --- | --- | --- | --- | --- | --- |
|  |  | %variance | p-value | %variance | p-value |
| Age_group | 2 | 1.486% | < 2.2e-16 | 4.764% | < 2.2e-16 |
| Gender | 1 | 0.717% | < 2.2e-16 | 1.509% | < 2.2e-16 |
| Year | 8 | 0.165% | < 2.2e-16 | 0.062% | 3.5e-15 |
| Mobility | 1 | 0.017% | 1.6e-03 | 0.022% | 4.6e-08 |
| Gender : Mobility | 1 | 0.042% | 6.2e-07 | n/a | n/a |
| Gender : Age_group | 2 | n/a | n/a | 0.064% | < 2.2e-16 |
| Age_group : Mobility | 2 | n/a | n/a | 0.006% | 1.3e-02 |
| Age_group : Year | 16 | n/a | n/a | 0.027% | 2.3e-03 |

*: First-time donation (7482 cases, 134,030 controls); Repeat (12,001 cases, 1,373,067 controls); df: degree of freedom; %variance: percent of the total phenotypic variance explained; underlined p-value of mobility in the Repeat donation model indicating insignificant additive effects; interacting was unlined to show terms were separated by “:”; Age_group: 1 (age<=22), 2 (22<age<=40), 3 (age>40).

Table S4: Odds ratios calculated for combinations of gender, age group and type of collection sites (fixed or mobile) using data between 2011 and 2019 *

| Term | First-time donation |  | Repeat donation |  |
| --- | --- | --- | --- | --- |
|  | OR (2.5% to 97.5%) | p-value | OR (2.5% to 97.5%) | p-value |
| Female_1_fixed | 1 |  | 1 |  |
| Female_1_mobile | 1.02 (0.94 to 1.11) | 0.664 | 0.85 (0.78 to 0.92) | 3.4e-05 |
| Female_2_fixed | 0.60 (0.53 to 0.66) | < 2.2e-16 | 0.35 (0.33 to 0.38) | < 2.2e-16 |
| Female_2_mobile | 0.57 (0.52 to 0.64) | < 2.2e-16 | 0.32 (0.30 to 0.35) | < 2.2e-16 |
| Female_3_fixed | 0.34 (0.28 to 0.40) | < 2.2e-16 | 0.15 (0.13 to 0.16) | < 2.2e-16 |
| Female_3_mobile | 0.34 (0.29 to 0.39) | < 2.2e-16 | 0.13 (0.12 to 0.14) | < 2.2e-16 |
| Male_1_fixed | 0.68 (0.60 to 0.77) | 7.9e-10 | 0.52 (0.46 to 0.58) | < 2.2e-16 |
| male_1_mobile | 0.55 (0.50 to 0.60) | < 2.2e-16 | 0.40 (0.36 to 0.45) | < 2.2e-16 |
| Male_2_fixed | 0.46 (0.41 to 0.51) | < 2.2e-16 | 0.21 (0.19 to 0.24) | < 2.2e-16 |
| male_2_mobile | 0.35 (0.31 to 0.40) | < 2.2e-16 | 0.19 (0.17 to 0.20) | < 2.2e-16 |
| Male_3_fixed | 0.22 (0.18 to 0.27) | < 2.2e-16 | 0.05 (0.04 to 0.06) | < 2.2e-16 |
| male_3_mobile | 0.19 (0.15 to 0.23) | < 2.2e-16 | 0.05 (0.05 to 0.06) | < 2.2e-16 |

*: First-time donation (7482 cases, 134,030 controls); Repeat (12,001 cases, 1,373,067 controls); OR (2.5% to 97.5%): odds ratio and 95% confidence intervals (i.e. 2.5% low end to 97.5% high end) in bracket; p-value: 2.2e-16 is the minimum p value adopted in the odds.ratio() function and anything lower is denoted as ‘< 2.2e-16’; Age_group: 1 (age<=22), 2 (22<age<=40), 3 (age>40).
